# Preparation of Ce- and La-Doped Li_4_Ti_5_O_12_ Nanosheets and Their Electrochemical Performance in Li Half Cell and Li_4_Ti_5_O_12_/LiFePO_4_ Full Cell Batteries

**DOI:** 10.3390/nano7060150

**Published:** 2017-06-20

**Authors:** Meng Qin, Yueming Li, Xiao-Jun Lv

**Affiliations:** 1 State Key Laboratory of Metastable Materials Science and Technology, College of Materials Science and Engineering, Yanshan University, Qinhuangdao 066004, China; qinbalele@163.com; 2 Key Laboratory of Photochemical Conversion and Optoelectronic Materials and HKU-CAS Joint Laboratory on New Materials, Technical Institute of Physics and Chemistry, Chinese Academy of Sciences, Beijing 100190, China; 3 Key Laboratory of Advanced Energy Materials Chemistry (Ministry of Education), Nankai University, Tianjin 300071, China

**Keywords:** lithium ion batteries, doping, anode, Li_4_Ti_5_O_12_, full cell

## Abstract

This work reports on the synthesis of rare earth-doped Li_4_Ti_5_O_12_ nanosheets with high electrochemical performance as anode material both in Li half and Li_4_Ti_5_O_12_/LiFePO_4_ full cell batteries. Through the combination of decreasing the particle size and doping by rare earth atoms (Ce and La), Ce and La doped Li_4_Ti_5_O_12_ nanosheets show the excellent electrochemical performance in terms of high specific capacity, good cycling stability and excellent rate performance in half cells. Notably, the Ce-doped Li_4_Ti_5_O_12_ shows good electrochemical performance as anode in a full cell which LiFePO_4_ was used as cathode. The superior electrochemical performance can be attributed to doping as well as the nanosized particle, which facilitates transportation of the lithium ion and electron transportation. This research shows that the rare earth doped Li_4_Ti_5_O_12_ nanosheets can be suitable as a high rate performance anode material in lithium-ion batteries.

## 1. Introduction

With the development of electric vehicles (EV), it is urgent to develop reliable batteries with high electrochemical performance and high safety, considering that commercial LiCoO_2_-graphite batteries have potential dangers due to the use of a graphite anode [1]. Thus, it is very important to develop reliable anode materials with high safety as well as high electrochemical performance.

Spinel Li_4_Ti_5_O_12_ (LTO) as a zero strain material has been regarded as an alternative anode material in Li-ion batteries (LIB)s with ideal safety performance. It features a flat high operation plateau potential (about 1.55 V versus lithium) [2,3], helping to prevent the formation of Li metal at low potential [4,5].

The key issue for Li_4_Ti_5_O_12_ is its poor intrinsic electronic conductivity (~10^−13^–10^−14^ S·cm^−1^) and low Li^+^ diffusion kinetics, which can restrict rate performance when LTO is applied in EV [2]. So far, a lot of strategies have been proposed to enhance the electronic conductivity and/or Li^+^ diffusion kinetics of LTO, including reducing the particle size [2,6,7,8,9,10,11,12,13,14], heteroatom doping, and coating with highly conductive additive [15,16,17,18]. The introduction of carbon materials can improve electron transfer on the surface of LTO, while lowering volumetric energy density [19,20,21,22,23]. Furthermore, the intrinsic electronic and ionic conductivities of LTO are not enhanced through conductive additive coating. Heteroatom doping of electrode materials has been provided a valid way for enhancing its intrinsic electronic and ionic conductivity according to previous reports [24,25,26,27]. For example, rare earth doping of Ce is able to improve cycling performance as well as rate performance due to the special electronic structure (a half full 4f electron shell) for of the rare earth metal [28,29,30]. It is reported that spherical La-doped LTO prepared by solid state synthesis displays good cycling performance compared to un-doped LTO; however, the rate performance can be further improved. 

High performance LTO must be developed to fulfill the ever-increasing requirement for batteries used in electric vehicles. Unsatisfactory rate performance is related to the large particle size of LTO (often larger than 1 mm) formed by solid-state synthesis and its poor intrinsic ionic conductivity [30,31,32,33,34,35].

In this report, we designed a route to prepare LTO by a combination of rare-earth doping and decreasing the particle size by synthesis of nanosheets. On one hand, the doping can enhance the intrinsic conductivity of LTO. On the other hand, doped LTO with nanosized particles help to decrease the diffusion path for electrons and Li ions. The Ce-doped LTO nanosheets were prepared by solvothermal synthesis followed by heat-treatment; La-doped LTO was also prepared for comparison. Electrochemical tests showed that the Ce- and La-doped LTO exhibited superior electrochemical performance as an anode in Li half cells and LTO/LiFePO_4_ (LFP) full cells.

## 2. Results and Discussion

**Figure 1:**
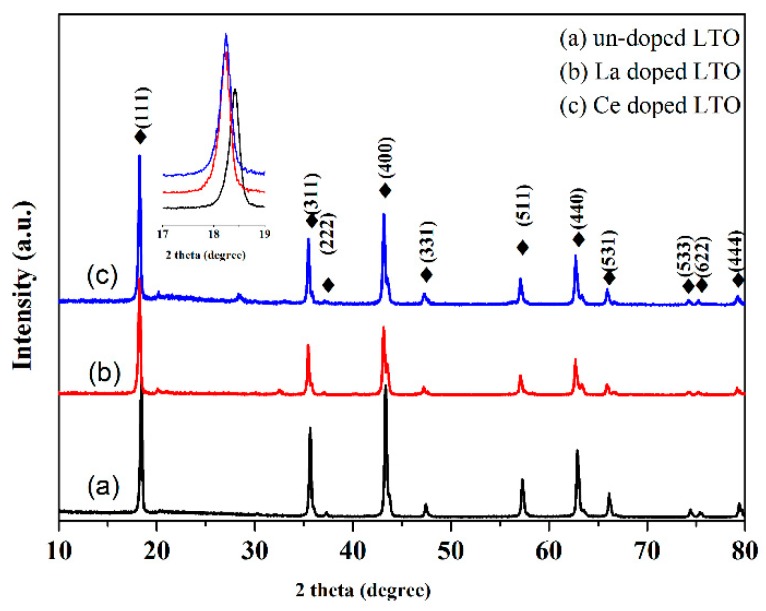
X-ray diffraction (XRD) patterns of pure Li_4_Ti_5_O_12_ (LTO), undoped LTO (**a**), La-doped LTO (**b**) and Ce-doped LTO (**c**).

Figure 1 presents the X-ray diffraction (XRD) patterns of pure LTO, La-doped LTO and Ce-doped LTO. For all three samples, the sharp and strong peaks were readily indexed into cubic spinel Li_4_Ti_5_O_12_ (JCPDS No. 72-426). The weight ratio of LTO in both samples was calculated to be ~95% according to Maud software [36]. The small peaks at 28 and 33° were related to minor CeO_2_. It can be observed that the (111) peak shifted to the left, which indicated the doping of La and Ce to LTO. 

It can be seen that the diffraction angle (111) decreased after Ce and La doping (inset of Figure 1), which indicated that the lattice parameters and lattice volume increased after doping. As is known, the radius of Ti^4+^ was smaller than those of La^3+^ and Ce^4+^; thus the introduction of La and Ce ions led to lattice volume expansion, suggesting the doping of La and Ce ions. The formula of Ce-LTO can be written as Li_4_Ti_5−x_Ce_x_O_12._ The cell parameter a for Ce- and La-doped LTO was almost the same (about 8.40 Å), while that of undoped LTO was only about 8.33 Å.

**Figure 2:**
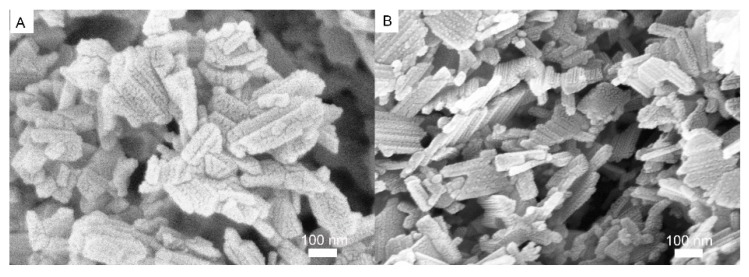
Scanning electron microscopy (SEM) images of undoped LTO (**A**) and Ce-doped LTO (**B**).

Figure 2A,B shows images of undoped LTO and Ce-doped LTO. As shown in Figure 2A, undoped LTO was composed of plenty of nanoparticles with various shapes. As shown in Figure 2B, the Ce-doped LTO showed a similar morphology while the particle size was within ~40 to 200 nm, a little smaller compared to that of the pure LTO (~50 to 250 nm). The morphology for La-doped LTO was similar to that of Ce-doped one. 

**Figure 3:**
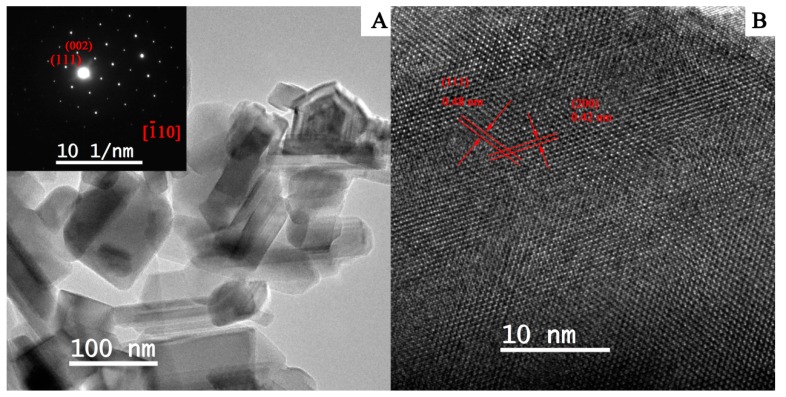
TEM image (**A**) and high resolution (HR)TEM image (**B**) of Ce-doped LTO [inset of A, the corresponding selected area electronic diffraction (SAED) pattern].

Transmission electron microscopy (TEM) was also used to further characterize the morphology of the Ce-doped LTO. Figure 3A discloses that the Ce doped LTO particles were composed of nanosheets with various shapes like rod and square, and the structure was very thin. The selected-area electron diffraction (SAED) pattern inset of Figure 3A displays the typical diffraction spots, indicating the single crystalline nature of cubic spinel phase in good agreement with the XRD results. The spaces of the perpendicular lattices for the Ce doped LTO derived from the high resolution (HR)TEM image were ~0.42 and 0.48 nm respectively, corresponding to the d-spacing of (002) and (111) planes of Li_4_Ti_5_O_12_.

**Figure 4:**
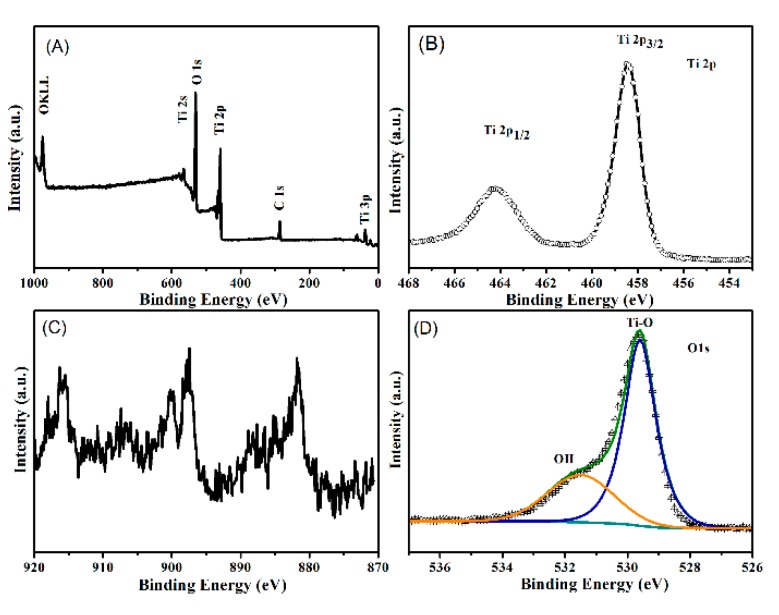
X-ray photoelectron spectroscopy (XPS) survey of Ce-doped LTO (**A**), Ti 2p (**B**), Ce 3d (**C**) and O 1s (**D**).

Figure 4 shows the X-ray photoelectron spectroscopy (XPS) spectrum of the Ce-doped LTO nanosheet. According to the survey spectrum (Figure 4A), the peaks assigned to Li, Ti, and O were observed in the as-prepared Ce-doped LTO.

Figure 4B shows the high-resolution XPS spectrum of Ti 2p. Two remarkable peaks were located at about 458.5 and 464.6 eV, corresponding to Ti 2p_3/2_ and Ti 2p_1/2_ of Ti^4+^, respectively. The O 1s spectrum of Ce-doped LTO is shown in Figure 4C. Figure 4C represents the high resolution spectrum of Ce. The peaks located at 916.3, 900.2, 897.6, 887.6 and 881.8 eV were attributed to the Ce^4+^ oxidation state, while those at 903.6, 887.6 eV corresponded to the Ce^3+^ oxidation state. The result indicates that both Ce^3+^ and Ce^4+^ species exist in Ce-doped LTO [37]. The dopants content of Ce element in doped LTO was less than 1% (atomic ratio) on the basis of the XPS result. The peak at 529.6 eV corresponded to the Ti–O bond in LTO, while the peak at 531.5 eV was assigned to OH.

**Figure 5:**
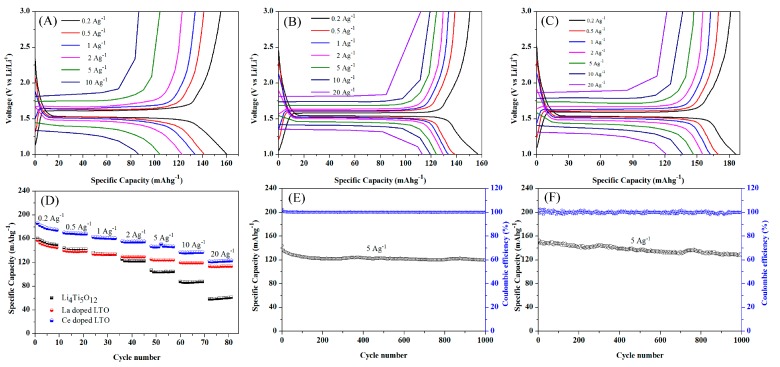
The galvanostatic discharge-charge profiles of pure LTO (**A**), La-doped LTO (**B**) and Ce-doped LTO (**C**) at varied current density; (**D**) Rate performance for these samples (**E**); Cycle performance plots of La-doped LTO (**E**) and Ce-doped LTO (**F**) at 5 Ag^−1^.

Figure 5 presents the charge/discharge profiles of undoped LTO, Ce-doped LTO and La-doped LTO from 0.2 to 20 Ag^−1^. As shown in the Figure 5A–C, the typical flat discharge plateaued at ~1.55 V and was clearly observed at lower rates for these samples. 

The pure LTO provided a specific capacity of ~159.7 mAh·g^−1^ at the current density of 0.2 Ag^−1^ for the initial discharge, while the doped LTO delivered a slightly higher value at the same rate. Even at higher rates, the electrodes had flat discharge plateaus. As shown in Figure 5D, both the La-doped LTO and Ce-doped electrodes delivered much higher capacity than undoped LTO, especially at higher rates, indicating a much improved rate performance after doping. For example, Ce-doped LTO and La-doped electrodes delivered a specific capacity as high as 123 and 113 mAh·g^−1^ at a current density of 20 Ag^−1^, respectively. As shown in Table 1, although the specific capacities at lower rates showed no obvious advantages over the other LTO electrodes reported previously, the discharge capacity at high rates showed obvious advantages (~147, 137 and 123 mAh·g^−1^ at 5, 10 and at 20 Ag^−1^ for Ce-doped LTO, respectively) over most previous reports on LTO based electrodes.

The cycling performance of Ce-doped and La-doped LTO electrodes at a high current density of 5 Ag^−1^ were studied for as many as 1000 cycles, as shown in Figure 5E,F. The capacitance maintained 86% of its initial specific capacity at 5 Ag^−1^ with a Coulombic efficiency of nearly 100% for both Ce- and La-doped LTO, showing excellent cycle stability for the rare earth doped LTO electrode. 

**Figure 6:**
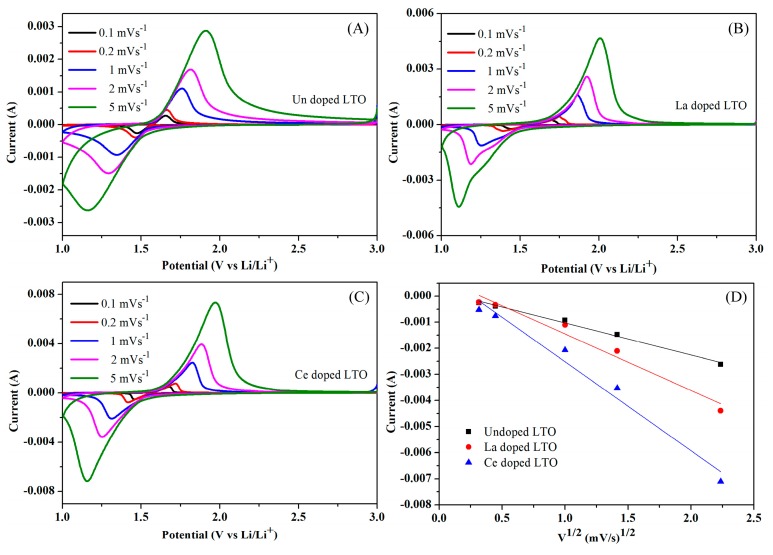
Cyclic voltammetry (CV) curves of undoped LTO (**A**); La-doped LTO (**B**); Ce-doped LTO (**C**); at varied scanning rates from 0.1 to 5.0 mV/s; The cathodic peak currents against square roots of scan rate (**D**).

Figure 6 displays the cyclic voltammogram of the undoped LTO, Ce-doped and La-doped LTO electrodes between 1 and 3 V (vs. Li^+^/Li) at a varied scan rate. The cathodic peaks for all samples were related to the Li-ions interaction into Li_4_Ti_5_O_12_, whereas the anodic peaks corresponded to the Li-ions de-intercalation from rock salt phase Li_7_Ti_5_O_12_ accompanied by Ti^4+^/Ti^3+^ redox reactions which were related to the following reaction:

$${\mathrm{Li}}_{4}{\mathrm{Ti}}_{5}O_{12}+3{\mathrm{Li}}^{+}+3e^{-}\overset{charge/discharge}{↔}{\mathrm{Li}}_{7}{\mathrm{Ti}}_{5}O_{12}$$

Notably, there was little change in lattice volume during the transition between the spinel to the rock-salt phase. Furthermore, both Ce- and La-doped LTO electrodes showed sharper and well-resolved peaks compared with the undoped electrodes at both low or high scan rates, indicating faster kinetics for the doped electrodes.

As shown in Figure 6D, the currents (Ip) of the cathodic peaks were observed to exhibit a linear relationship to the square root of the scanning rate (ν^1/2^), indicating a diffusion-controlled mechanism [43]. Obviously, the slope of cathodic peaks for the Ce-doped LTO was higher than that of the undoped one, indicating a higher diffusion coefficient for the doped samples. The Randles-Sevchik equation was used to evaluate the diffusion coefficient for the diffusion controlled reactions:

D = (Ip/2.686 × 10^5^ n^3/2^ A C ν^1/2^)^2^ (1)

where D represents the diffusion coefficient, Ip is peak current, n is the charge transfer number, A is the surface area of the electrode, C is the bulk concentration of the ions in the electrode, and ν represents the scan rate. According to Equation (1), the apparent diffusion coefficient of Li ions for Ce- and La-doped LTO electrodes was calculated to be approximately 1.55 × 10^−10^ and 5.94 × 10^−11^ cm^2^·s^−1^ at 5 mV·s^−1^ respectively, which was considerably higher than undoped LTO (2.11 × 10^−11^ cm^2^·s^−1^) at the same scan rate. These results demonstrated that the rare earth doping can effectively improve the electrochemical reaction kinetics of Li^+^ insertion/de-intercalation.

The superior electrochemical performance for rare earth-doped LTO nanosheets can be ascribed to the following factors. One hand, the rare-earth doping is able to enhance the intrinsic conductivity of LTO due to the changing band gap [30]. Although the solid electrolyte interface (SEI) protective film does not form during the charge/discharge process due to the high operation potential of ~1.55 V, the interfacial reactions between LTO and electrolytes consume Li and O from LTO during long-term cycles, leading to the decay of the LTO electrode [44,45].

**Figure 7:**
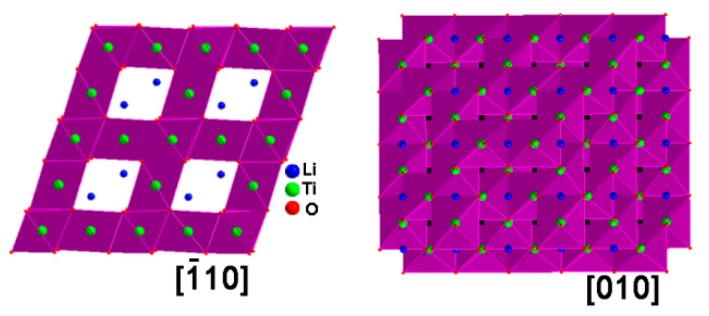
LTO crystal structure view from [$\bar{1}$10] and [010].

On the other hand, the nanoscale particle size for rare earth-doped LTO helps to decrease the transport distance for both Li^+^ and electrons, and facilitate close contact between the electrode and electrolyte, which can lower concentration polarization and improve the rate performance. For the spinel-based LTO, the diffusion rate of a Li atom across the [110] direction (or equivalent direction, such as [101], [011] and [$\bar{1}$10] is much faster than other direction such as [010], due to the large tunnel structure in this direction, shown in Figure 7. [46] As seen from Figure 3, LTO nanosheets in as-prepared composites grow along [110], facilitating the fast diffusion of the Li ion.

**Figure 8:**
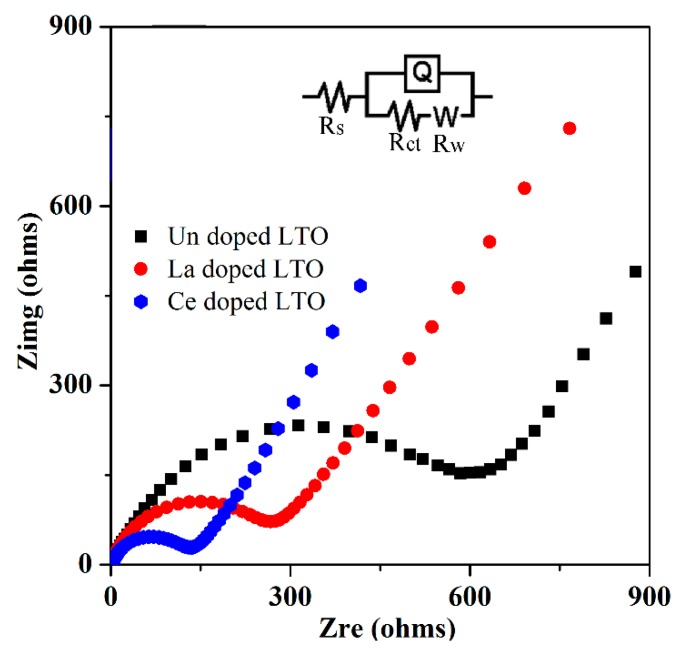
Electrochemical impedance spectra of Ce/La-doped LTO, undoped LTO and pure Li_4_Ti_5_O_1__2_ electrodes (inset shows the equivalent circuit).

Electrochemical impedance spectroscopy (EIS) was also used to further measure the electrochemical performance for these three samples. As shown in Figure 8, the typical semicircles in the middle-high frequency were observed, which presents a charge transfer resistance. The semicircle of the doped LTO electrode was much smaller than that of undoped LTO, indicating a smaller charge transfer resistance after doping.

The charge transfer resistance according to the fitting circuit (inset of Figure 8) was about 108 ohms, which was much smaller than those of undoped LTO electrodes (230 and 530 ohms, respectively.) This experiment also showed that the ionic conductivity of LTO could be enhanced by rare earth heteroatom doping.

The LTO/LiFePO_4_ full cell is considered to be the safest Li ion battery, due to the high plateau of LTO and the high thermal stability of LiFePO_4_ and LTO. 

**Figure 9:**
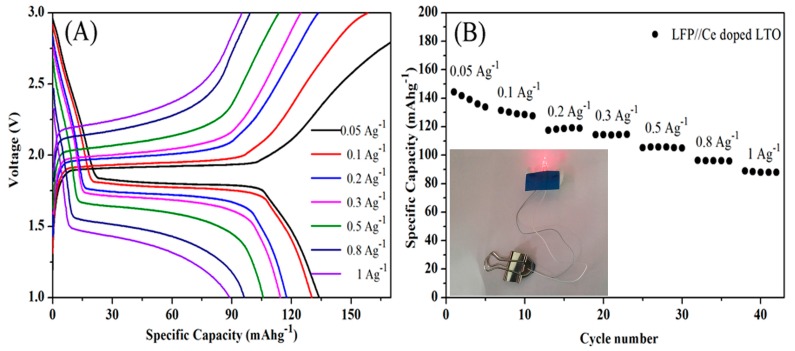
(**A**) Galvanostatic charge-discharge voltage profiles of a LiFePO_4_/Ce-doped LTO full cell at varied current densities within a voltage of 1–3 V; (**B**) The rate performance for LiFePO_4_/Ce doped LTO full cell (inset: digital picture of a light-emitting diode (LED) powered by an as-fabricated full cell).

Figure 9A shows the voltage profiles of a LiFePO_4_/Ce-doped LTO full cell at varied current densities. A long and flat discharge plateau potential was clearly observed at about 1.4–1.8 V at different current densities, similar to a previous report by Zaghib et al. [47]. The decreased discharge plateau at high current densities can be attributed to the kinetics limited by ion diffusion within the electrolyte and the electrode/electrolyte interface.

The LiFePO_4_/Ce-doped LTO full cell also showed excellent rate performance, as shown in Figure 9B. The full cell was capable of delivering a specific capacity of 119 mAh·g^−1^ at 0.2 Ag^−1^, which was much higher than the recently reported value based on LTO/LFP full cells [47,48]. It can deliver a specific capacity of 88 mAh·g^−1^ even at 1 Ag^−1^. A white LED can be powered using as fabricated full cell, as shown in inset of Figure 9B.

## 3. Materials and Methods

### 3.1. Materials

All reagents were analytical-grade and used without further purification except otherwise stated. 

#### Synthesis of Ce- and La-Doped LTO Nanosheets

Ce-doped LTO nanocomposites were prepared using a similar method to previous reports [6,30,39]. The procedure was as follows: Three milliliters of tetrabutyltitanate (Tianjin Kermel analytical reagent Co., Tianjin, China), 0.015 g of CeCl_3_·7H_2_O (Alfa-Aesar Chemicals Co., Shanghai, China), and 0.41 g of LiOH·H_2_O (Sinopharm Chemical Reagent Co., Shanghai, China) were mixed in 20 mL of ethanol at room temperature. Then, 25 mL of deionized water were added under stirring, and incubated for 4 h. Then, the mixture was then put into a Teflon-lined stainless autoclave and heated at 180 °C for 48 h. The white precipitate was filtered, washed with ethanol for several times and dried at 80 °C for ~12 h. Finally, the white powder was heated at 700 °C for 6 h in a tube furnace with air, to obtain the Ce-doped LTO. La-doped samples were prepared similar to the above procedure except LaCl_3_ (Alfa-Aesar Chemicals Co., Shanghai, China) was added in place of CeCl_3_. For comparison, undoped LTO was prepared similar to the above procedure, with no LaCl_3_ or CeCl_3_ added. 

### 3.2. Characterization

X-ray diffraction (XRD) measurements of the powder samples were recorded on a D-max 2500 X-ray powder diffractometer using a graphite monochromator with Cu Kα radiation (λ = 1.5406 Å), with a scattering angles from 10° to 80° at a scanning rate of 4° min^−1^. Scanning electron microscopy (SEM) was performed on a Hitachi S-4800. TEM, HRTEM, and SAED were conducted on the FEI Tecnai F20 G2 S-TWIN. XPS was recorded the on ESCALAB 250XI, and the binding energy was calibrated with C1s = 284.8 eV.

### 3.3. Electrochemical Measurements

The electrodes were constructed by mixing the active materials (80 wt. %), Super P conductive (15 wt. %) (Sigma-Aldrich Chemicals Co., Shanghai, China) and polyvinylidene fluoride (PVDF) (Alfa-Aesar Chemicals Co., Shanghai, China) binder (5 wt. %) uniform uniformly in N-methyl pyrrolidinone (NMP) (Alfa-Aesar Chemicals Co., Shanghai, China) solvent and spreading the mixture onto Cu foil. The electrode was dried under a vacuum at 120 °C for 12 h. The mass loading for the active materials in the as-fabricated electrode was about 1mg·cm^−2^. The half cell was assembled inside an argon-filled glove box (Braun, H_2_O < 0.5 ppm and O_2_ < 0.5 ppm). Lithium metal foil was used as the counter and the reference electrode. A commercial (1 M) LiPF_6_ electrolyte was used in the cell, and commercial polypropylene (Celgard 2400) was used as the separator. The galvanostatic charge/discharge tests of the assembled cells were carried out on a Arbin battery testing unit at varied current densities between the voltage limits of 1.0 and 3.0 V (vs. Li^+^/Li). The theoretic capacity of LTO is 175 mAh·g^−1^; thus 1C was defined as 175 mA·g^−1^ for LTO. CV measurements were recorded between the potential range of 1.0 and 3.0 V (vs. Li^+^/Li) on a P4000 electrochemical workstation (Princeton Applied Research, Oak Ridge, TN, USA) at varied scanning rates. EIS was also measured by a P4000 electrochemical workstation within a frequency range of 0.1–10^5^ Hz at the amplitude of 5 mV versus the open circuit potential.

A CR2032 coin cell was used to assemble the full cell, and the full cell was anode-limited. The commercial LiFePO_4_ electrode pasted onto Al foil was used as a cathode, while Ce-doped LTO pasted on Cu foil was used as an anode. The mass loading of LiFeO_4_ was considerably higher than that of Ce-doped LTO, and the capacity was calculated based on the mass of Ce-doped LTO. The separator and electrolyte were the same as those in the half cell. The LTO/LiFePO_4_ full cells were charged/discharged by a constant current method within a potential range of 1 and 3 V. 

## 4. Conclusions

In conclusion, Ce- and La-doped LTO nanosheets were successfully prepared via a facile method. Through a combination of rare earth doping and decreasing the particle size of LTO, the as-prepared doped LTO nanosheets showed superior electrochemical performance, especially in high rate performance and cycling stability, both in Li half cells and LTO/Li_4_FePO_4_ full cells. Considering the excellent electrochemical performance, as-prepared rare earth doped sample showed a promising future in lithium ion batteries with a requirement of high safety and high rate performance. Furthermore, this method may extend to other electrode materials to modify their electrochemical performance.

## Figures and Tables

**Table 1:** Comparison of the electrochemical performance for the LTO electrode based on the Li-half cell.

Electrode Materials	Specific Capacity (mAh·g^−1^)	Current Density (A·g^−1^)	References
Li_4_Ti_5_O_12_@C nanotube	81	17.5	[38]
N-Carbon-coated LTO	129	1.75	[20]
Rutile-coated LTO	110	10.5	[31]
LTO nanowire arrays	118	5.25	[13]
Carbon-coated LTO	110	3.5	[39]
Cr-doped LTO	120	1.75	[40]
Mesoporous LTO@C	62	14	[41]
Gd-doped LTO/TiO_2_	111	20	[42]
Ce^3^^+^-doped LTO	105.2	1.75	[17]
Ce-doped LTO/C	145.3	1.75	[33]
La-doped LTO	113.8	8.75	[28]
Ce-doped LTO	155 147 137 123	2 5 10 20	This work
La-doped LTO	130 125 120 113	2 5 10 20	This work
